# Gender differences in neurocognitive assessments: insights from a pilot study with the International Neurocognitive Test Profile (INCP) digital battery

**DOI:** 10.1007/s40211-024-00510-6

**Published:** 2024-09-24

**Authors:** Bernd Maierhofer, Daria Grigoryeva, Beatrice Beck, Johann Lehrner

**Affiliations:** 1 Department of Neurology, Medical University of Vienna, Währinger Gürtel 18–20, 1090 Vienna, Austria; 2 Faculty of Psychology, University of Vienna, Vienna, Austria

**Keywords:** Dementia screening, Digital testing, Digital cognitive assessment, Subjective cognitive decline, Sex differences, Demenz-Screening, Digitale Tests, Digitales kognitives Assessment, Subjektive kognitive Störung, Geschlechtsunterschiede

## Abstract

**Background:**

The aging global population has led to an increase in the number of dementia diagnoses, with projections indicating a continued upward trend. This demographic change presents profound challenges for patients, their families, and healthcare systems worldwide. Consequently, the demand for reliable and user-friendly screening tools that can detect dementia at early stages and monitor its progression is more critical than ever. The International Neurocognitive Test Profile (INCP), developed at the Medical University of Vienna, aims to address this need by offering a digital test battery for the early detection of dementia. This study forms a part of the INCP’s ongoing development and evaluation, specifically investigating the influence of gender on test outcomes.

**Methods:**

Seventy participants, recruited through flyers at the Vienna General Hospital, completed the INCP assessment using tablets as part of the study. The effect of gender on performance across various INCP subtests was analyzed using Mann–Whitney *U* tests. For further exploratory analysis, a correlation matrix was calculated encompassing demographic variables (age and education), screening data, and all INCP subtests.

**Results:**

The analysis revealed significant gender differences in two INCP subtests related to executive functions. Males outperformed females on the Figure Fluency Test (*r* = 0.30, indicating a moderate effect) and the Dice 2‑*n *Back Test (*r* = 0.29, indicating a small effect). However, when correcting for multiple comparisons, no significant gender disparities were observed in the scores of the subtests.

**Conclusion:**

The identification of possible gender differences in specific subtests underscores the importance of considering gender as a variable in the further development and evaluation of the INCP. These findings offer valuable insights for the design and planning of future studies involving the INCP.

## Abstract (de)

**Hintergrund:**

Die alternde Weltbevölkerung hat zu einem Anstieg der Demenzdiagnosen geführt. Dieser demografische Wandel stellt Patienten, ihre Familien und Gesundheitssysteme weltweit vor tiefgreifende Herausforderungen. Daher ist die Nachfrage nach zuverlässigen und benutzerfreundlichen Screening-Tools, die Demenz in frühen Stadien erkennen und ihr Fortschreiten überwachen können, wichtiger denn je. Das International Neurocognitive Test Profile (INCP) ist eine digitale Testbatterie zur Früherkennung von Demenz. Diese Studie ist Teil der fortlaufenden Entwicklung und Evaluierung des INCP und untersucht insbesondere den Einfluss des Geschlechts auf Testergebnisse.

**Methoden:**

Siebzig Teilnehmende wurden für diese Studie rekrutiert. Der Einfluss des Geschlechts auf die Leistung in verschiedenen INCP-Untertests wurde mithilfe von Mann-Whitney-U-Tests analysiert. Für die weitere explorative Analyse wurde eine Korrelationsmatrix berechnet, die demografische Variablen (Alter und Bildung), Screening-Daten und alle INCP-Untertests umfasst.

**Ergebnisse:**

Die Analyse ergab signifikante Geschlechtsunterschiede in zwei INCP-Untertests im Zusammenhang mit exekutiven Funktionen. Männer übertrafen Frauen beim Figure Fluency Test (r = 0,30, was auf einen moderaten Effekt hinweist) und beim Dice 2‑n Back Test (r = 0,29, was auf einen kleinen Effekt hinweist). Nach Korrektur für multiples Testen wurden keine signifikanten geschlechtsspezifischen Unterschiede der Untertests festgestellt.

**Schlussfolgerung:**

Die Identifizierung von möglichen Geschlechtsunterschieden in bestimmten Subtests unterstreicht die Bedeutung der Berücksichtigung des Geschlechts als Variable bei der Weiterentwicklung und Bewertung des INCP. Diese Ergebnisse bieten wertvolle Erkenntnisse für die Gestaltung und Planung zukünftiger Studien der INCP.

Dementia, characterized by its steadily increasing prevalence, poses significant challenges to individuals, healthcare infrastructure, and societal welfare. According to the World Health Organization, the global incidence stood at 55 million cases in 2023, with Austria alone accounting for 100,000 cases, as indicated by the Austrian Alzheimer Association. Projections suggest a further increase in these numbers, attributable to the upward trend in life expectancy [1, 2].

This surge in dementia cases, coupled with heightened public consciousness of the condition, has led to an increase in consultations with healthcare providers by individuals experiencing cognitive symptoms, identified either personally or by observers. Subjective cognitive decline (SCD) represents a condition where individuals notice a decline in their cognitive abilities, yet clinical assessments do not corroborate these observations, positing SCD as a potential precursor to dementia. Nonetheless, the nuance of cognitive alterations in the early stages presents considerable diagnostic challenges, complicating the differentiation between SCD sufferers and cognitively healthy individuals [3]. The tools currently available for the early detection of cognitive impairment are not only scarce but also tend to be costly and demand significant time investments, requiring recurrent visits to memory clinics or similar healthcare facilities, with the added limitation of testing in nonfamiliar settings. Consequently, there is a critical need for a simple, at-home cognitive evaluation tool to facilitate widespread cognitive screening [4].

The advancement of computerized cognitive assessments has been a focus for years, driven by the escalating incidence of dementia and the consequent demand for more economical, efficient evaluation tools that can be seamlessly incorporated into clinical practices [5]. Nevertheless, the utilization of tablets for testing introduces an innovative alternative to the conventional computerized tests that require a mouse, as well as to the traditional pen-and-paper assessments. The potential of digital cognitive evaluations is significant, with evidence suggesting that these types of test batteries can yield dependable outcomes characterized by commendable sensitivity and specificity [6–8].

The International Neurocognitive Test Profile (INCP) represents a comprehensive digital test battery designed to track cognitive decline and monitor its progression over time through recurrent assessments. In the course of its development, this research aims to ascertain the influence of gender on the psychometric outcomes of the INCP in a pilot study setting.

The investigation focuses on determining whether a gender-based advantage exists within various cognitive domains, including complex attention, learning and memory, executive function, language, social cognition, and perceptual–motor skills [9]. The literature offers a nuanced perspective on the influence of gender and biological distinctions on memory, with evidence suggesting that women may have a superior capacity in certain memory tasks compared to men [10–12]. These effects extend across various cognitive domains, displaying a varied pattern. Research indicates a male advantage in specific elements of executive functions, notably in visual–spatial working memory, whereas women have been observed to show greater proficiency in tasks related to object location memory [13, 14]. Moreover, studies have identified a female advantage in language skills and social cognitive abilities [11, 12]. It is important to note that previous investigations into the impact of gender on cognitive testing have predominantly focused on isolated assessments targeting single cognitive domains.

In prior research, gender has been shown to influence performance on two specific subtests within the INCP, which evaluate semantic memory and incidental learning, namely the Capital Knowledge and Flag Knowledge tests [4]. Heidinger and Lehrner [4] reported that male participants outperformed female participants in the memory aspects of these tasks. Although these particular assessments were not incorporated into the current study, other tests examining learning and memory were explored. Subsequent research assessing the role of educational attainment and age on the INCP outcomes has revealed that tests measuring memory, complex attention, and executive function are significantly impacted by educational level, whereas an increase in age was associated with decreased performance on the Digit Symbol Test (DST), a critical assessment of complex attention [15, 16].

This investigation aims to examine the influence of gender on the performance of various subtests within the INCP. Given the ongoing development and evaluation of the INCP, data pertaining to age and educational background are limited. Consequently, this study also seeks to assess the effects of demographic factors, such as age and education, on the cognitive performance measured by the INCP.

## Methods

### Sample

This pilot study included 70 healthy participants recruited through public advertisements. Prior to participation, written consent was obtained from all participants. Eligibility for the study was determined based on the absence of neurological disorders, psychiatric diagnoses, dementia, and other conditions that could impair cognitive function. A screening process was employed to ensure adherence to these criteria. Initially, participants undertook the Vienna Visuo-Constructional Test (VVT 3.0), a validated instrument for identifying indicators of cognitive decline [17]. Only those who scored 9 points or above were included. Additionally, the Wortschatztest (WST) was administered to evaluate verbal intelligence, requiring a minimum WST-IQ score of 85 for participation. The Beck Depression Inventory II (BDI-II) was also utilized to assess depressive symptoms, with a cutoff score of 13 or below as a criterion for inclusion.

### Measures

The INCP, developed at the Medical University of Vienna, is a comprehensive digital battery designed to assess five of the six cognitive domains outlined in the Diagnostic and Statistical Manual of Mental Disorders, Fifth Edition, through a series of subtests [9]. This study is part of the ongoing evaluation of the INCP’s third and latest version.

Following the provision of written consent and completion of the VVT 3.0, participants proceeded to undertake the INCP assessments on an Apple iPad Air 2. The subtests were presented in a predetermined sequence, allowing participants to progress at their own pace under the guidance of a test administrator who was present to clarify any questions. Owing to the INCP’s continuous development, the study did not encompass all available subtests.

In the DST, participants were required to match various symbols with numbers according to a specified template. This test consisted of three rounds, each lasting 60 s, with the objective being to identify as many correct pairs as possible within the established time.

The Face Identification Test (FACE) involved recognizing 16 celebrities from black-and-white images, with four name options provided for each photograph.

Subsequently, during the City Identification Test (CITY), participants matched capital cities to their respective countries from a set of four country options, focusing on the correct city–country pairings.

The next assessment, the Pattern Cancel Test (PCT), displayed a target pattern alongside a series of similar and distinct patterns. The objective was to find all identical matches within a three-minute timeframe.

In the Emotion Face Test–short (EFT-s), which explores emotional recognition, participants were presented with a sequential array of faces, each characterized by mouth corners oriented either upwards or downwards. This evaluation was structured into two segments, with 30 faces per round. Within these segments, participants were provided with two options for response: a “happy” button to indicate faces with upward-facing mouth corners and a “sad” button for those with downward-facing corners. In the subsequent round, the response protocol was inverted.

Turning to linguistic skills, in the Verbal Vocabulary Test (VVT), the task was to determine whether a displayed word was an actual German word, as recognized by the Duden German language dictionary. Participants were provided with a list consisting of 50 genuine and 50 similar fictitious words. The decision was made by selecting either a “Yes” or a “No” button.

In the context of decision-making under time constraints, the Traffic Light Test–short (TLT-s) displayed a traffic light alternating between a green and a red light. This test was organized into two rounds, with the traffic light illuminating 30 times per round. Each illumination lasted for two seconds, with intervals of one second in between. Participants were required to press a “Go” button when the green light was shown and a “Stop” button upon the appearance of the red light. The instructions for the second round were reversed.

In the Figure Fluency Test (FFT), the objective was to create as many distinct shapes as possible within a three-minute period. The shapes were formed by connecting lines on a grid composed of dots.

During the Dice 2‑*n* Back (DICE) task, four dice were displayed, moving from right to left. The die on the far left was obscured, and the second die from the right was highlighted. As the dice progressed across the screen, participants were to press the “Equal” button if the highlighted die showed the same number of points as the obscured die and the “Unequal” button if the numbers differed.

Lastly, in the Time Duration Test (TDT), 18 shapes were shown one after another for varying lengths of time. Participants were required to estimate the duration for which each shape was displayed and select it when it reappeared for the identical duration.

### Statistical analysis

The statistical analysis was carried out using SPSS Statistics 27 (IBM, Armonk, NY, USA). Given the nonnormal distribution of the data, Mann–Whitney *U* tests were utilized to evaluate the impact of gender on the subtest scores of the INCP. Furthermore, effect sizes were derived from the results of the Mann–Whitney *U* tests according to the following formula:

$$r=\left| \frac{Z}{\sqrt{N}}\right|$$

Additionally, a correlation matrix incorporating the INCP subtests, demographic information, and screening data was computed. This analysis aimed to determine whether subtests assessing the same cognitive domain yielded correlated scores and to further investigate variables influencing the psychometric properties observed within the INCP dataset.

Considering that the INCP is in an ongoing developmental stage and possesses a relatively brief history of application, the threshold for statistical significance was established at *α* = 0.05. In line with the parameters suggested by Cohen [18], effect sizes were categorized as follows: *r* < 0.3 indicating a small effect, 0.3 ≤ *r* < 0.5 indicating a moderate effect, and *r* ≥ 0.5 denoting a large effect.

## Results

The study’s cohort comprised 70 participants, of which 25 (35.7%) were male and 45 (64.3%) were female. The average age of the participants was 41.60 years, exhibiting a standard deviation (SD) of 17.38 years. The range of educational attainment was 9–27 years, with an average of 16.40 years and a standard deviation (SD) of 3.99 years. The participants achieved an average score of 9.84 (SD = 0.37) on the VVT 3.0, a mean score of 109.23 (SD = 8.00) on the WST-IQ, and a mean score of 4.59 (SD = 3.73) on the BDI-II, as detailed in Table 1.

**Table 1:** Demographic and screening data

	Mean	SD	Range
Age	41.60	17.38	19–80
Gender	0.64	0.48	0–1
Years of education	16.40	3.99	9–27
VVT 3.0	9.84	0.37	9–10
WST-IQ	109.23	8.00	90–129
BDI-II	4.59	3.73	0–13

*SD* standard deviation, *BDI-II* Beck Depression Inventory II, *WST-IQ* Wortschatztest, *VVT 3.0* Vienna Visuo-Constructional Test

Owing to the nonnormal distribution of data from the INCP subtests, Mann–Whitney *U* tests were employed to evaluate gender-related differences in subtest performance. These analyses were performed individually for each subtest, with effect sizes subsequently determined. Notably, significant disparities were observed in two subtests. In the DICE task, male participants (median = 49) demonstrated superior performance compared to female participants (median = 46.50). This difference was evidenced by a Mann–Whitney *U* statistic of 296.00 (*Nm* = 21, *Nf* = 44), a *Z-*score of −2.37, a *p*-value of 0.02, and an effect size *r* of 0.29, indicative of a small effect. Similarly, in the FFT, male participants (median = 22.00) outperformed their female counterparts (median = 15.50), supported by a Mann–Whitney *U* value of 317.00 (*Nm* = 23, *Nf* = 44), a *Z*-score of −2.50, a *p*-value of 0.01, and an effect size *r* of 0.30, denoting a moderate effect. However, when adjusting for multiple comparisons using Bonferroni correction, dividing α by the number of tests carried out [12], the critical value for individual tests is *α* = 0.004. Hence, when correcting for multiple comparisons, none of the examined subtests showed significant differences. The results of the nonparametric tests and the effect sizes are listed in Table 2.

**Table 2:** Median of the subtest scores for male and female participants with interquartile ranges in parentheses and results of Mann–Whitney *U* tests with Mann–Whitney *U* statistic (U), Z‑value (Z), two-tailed *p*-value (*p*), and effect sizes

		Median						
	*N*	Total	Male	Female	U	Z	*p*	Effect size
*CITY*	70	13.00 (2.00)	14.00 (3.00)	13.00 (3.00)	411.50	−1.87	0.06	0.22
*FACE*	70	12.00 (5.00)	11.00 (5.00)	13.00 (7.00)	513.50	−0.61	0.55	0.07
*EFT 1*	68	29.50 (2.00)	29.00 (2.00)	30.00 (2.00)	497.50	−0.28	0.78	0.03
*EFT 2*	68	28.00 (4.00)	28.00 (4.00)	28.00 (4.00)	513.50	−0.05	0.96	0.01
*VVT*	38	84.00 (6.25)	84.00 (6.25)	84.50 (6.25)	132.00	−0.77	0.46	0.12
*TLT 1*	66	30.00 (2.00)	30.00 (2.00)	30.00 (2.00)	462.00	−0.33	0.75	0.04
*TLT 2*	66	29.00 (2.00)	30.00 (2.00)	29.00 (2.00)	380.00	−1.50	0.13	0.19
*PCT*	70	14.00 (3.00)	14.00 (3.00)	14.00 (3.00)	515.00	−0.59	0.56	0.07
*TDT*	65	37.04 (10.37)	37.04 (6.53)	37.01 (11.72)	329.00	−0.98	0.33	0.12
*DICE*	64	48.00 (7.50)	49.00 (3.00)	46.50 (12.00)	296.00	−2.37	0.02	0.29
*FFT*	67	18.00 (16.00)	22.00 (18.00)	15.50 (15.75)	317.00	−2.50	0.01	0.30
*DST*	58	73.00 (18.25)	70.00 (23.00)	73.00 (18.00)	399.50	−0.05	0.962	0.01

*CITY* City Identification Test, *FACE* Face Identification Test, *EFT* Emotion Face Test, *VVT 3.0* Vienna Visuo-Constructional Test, *TLT* Traffic Light Test, *PCT* Pattern Cancel Test, *TDT* Time Duration Test, *DICE* Dice 2‑*n* Back, *FFT* Figure Fluency Test, *DST* Digit Symbol Test

Furthermore, a correlation matrix was calculated to examine the relationship between demographic and screening variables and the performance on INCP subtests, as well as to investigate correlations within subtests evaluating the same cognitive domains. Spearman’s rank correlation coefficients were applied for this matrix, with the findings documented in Table 3.

**Table 3:** Spearman correlation matrix of demographic data, screening measures, and the subtests of the International Neurocognitive Test Profile (INCP), with correlation coefficients and significance levels

	CITY	FACE	EFT 1	EFT 2	VVT	TLT 1	TLT 2	PCT	TDT	DICE	FFT	DST
*Age*	0.19	0.74^***^	−0.49^***^	−0.37^**^	0.34^*^	< 0.01	−0.22	−0.29^*^	0.10	−0.57^***^	−0.43^***^	−0.62^***^
*Gender*	−0.23	0.07	−0.03	0.01	−0.12	−0.04	−0.19	−0.07	0.12	−0.30^*^	−0.31^*^	−0.01
*Edu*	0.48^***^	−0.05	0.13	0.21	0.40^*^	0.23	0.22	0.03	−0.19	0.12	0.06	0.21
*VVT 3.0*	0.03	−0.33^**^	< 0.01	0.06	−0.19	−0.14	−0.05	0.10	0.06	0.11	0.01	0.18
*BDI-II*	−0.17	0.15	−0.21	−0.46^***^	−0.15	0.05	0.11	0.05	0.25^*^	−0.17	−0.30^*^	−0.12
*WST-IQ*	0.30^*^	0.08	0.20	0.13	0.57^***^	0.01	0.19	0.06	−0.20	0.13	0.13	0.01
*FACE*	0.25^*^	1.00	−0.27^*^	−0.31^*^	0.30	0.08	−0.10	−0.24^*^	−0.03	−0.47^***^	−0.27^*^	−0.42^***^
*EFT 1*	−0.13	−0.27^*^	1.00	0.32^**^	0.16	0.17	0.17	0.16	−0.14	0.48^***^	0.24^*^	0.38^**^
*EFT 2*	0.04	−0.31^*^	0.32^**^	1.00	0.01	0.19	0.23	0.15	−0.28^*^	0.24	0.27^*^	0.30^*^
*VVT*	0.19	0.30	0.16	0.01	1.00	0.58^***^	0.20	−0.10	−0.22	0.05	−0.06	−0.05
*TLT 1*	0.11	0.08	0.17	0.19	0.58^***^	1.00	0.17	−0.03	−0.24	0.14	−0.08	0.01
*TLT 2*	0.07	−0.10	0.17	0.23	0.20	0.17	1.00	0.04	−0.07	0.06	0.22	0.18
*PCT*	−0.07	−0.24^*^	0.16	0.15	−0.10	−0.03	0.04	1.00	0.01	0.23	0.16	0.37^**^
*TDT*	−0.04	−0.03	−0.14	−0.29^*^	−0.22	−0.24	−0.07	0.01	1.00	−0.17	−0.06	−0.28^*^
*DICE*	−0.18	−0.47^**^	0.48^***^	0.24	0.05	0.14	0.06	0.23	−0.17	1.00	0.26^*^	0.48^***^
*FFT*	0.08	−0.27^*^	0.24^*^	0.27^*^	−0.06	−0.08	0.22	0.16	−0.06	0.26^*^	1.00	0.21
*DST*	0.15	−0.42^***^	0.38^**^	0.30^*^	−0.06	0.01	0.18	0.37^**^	−0.28^*^	0.48^***^	0.21	1.00

*Edu *education*, BDI-II* Beck Depression Inventory II, *WST-IQ* Wortschatztest, *CITY* City Identification Test, *FACE* Face Identification Test, *EFT 1/2* Emotion Face Test round 1/2, *VVT 3.0* Vienna Visuo-Constructional Test, *TLT 1/2* Traffic Light Test round 1/2, *PCT* Pattern Cancel Test, *TDT* Time Duration Test, *DICE* Dice 2‑*n* Back, *FFT* Figure Fluency Test, *DST* Digit Symbol Test

* significant to a significance level of 0.05, ** significant to a significance level of 0.01, *** significant to a significance level of 0.001

## Discussion

This study sought to assess the influence of gender on the performance scores within the subtests of the INCP. Employing Mann–Whitney *U* tests for statistical analysis revealed that gender significantly impacted the outcomes of the FFT and DICE tasks, with the FFT displaying a moderate effect. Males exhibited superior performance in tasks assessing executive function. Although prior research has indicated a male advantage in executive function [13], the literature presents a mixed view on gender’s role in this cognitive domain [14]. No other INCP subtests demonstrated significant differences between male and female participants, indicating no definitive gender advantage across the remaining cognitive domains. This finding diverges from previous studies that suggest varying levels of performance between genders in memory and learning tasks [4, 10, 11], as well as reports of a female advantage in language-related tasks, though such an effect was not observed in the VVT within this study [12]. However, it has to be noted that when correcting for multiple comparisons neither the FFT nor the DICE test show significant differences between male and female participants.

An exploratory analysis revealed correlations between age and education levels and performance across multiple INCP subtests, aligning with findings from earlier studies [15, 16].

Age demonstrated correlations across all five assessed cognitive domains. In alignment with existing research, a negative correlation was observed between age and performance on two executive function subtests: the FFT and DICE [19, 20]. Additionally, age was inversely related to outcomes on the complex attention task, the DST, and showed a positive association with the language task, the VVT. Notably, previous studies have documented an age-associated decline in language abilities, such as vocabulary recall [21]. A reduction in social cognition abilities with advancing age was inferred from the negative correlation with the EFT‑s, corroborating literature that suggests a diminishing capacity for emotion recognition beginning in middle age [22]. An exception was noted in the FACE, a memory assessment subtest, where older participants displayed a relative advantage. This phenomenon may be attributed to the selection of celebrities for this task, as the featured actors and musicians are presumably more familiar to older demographics. These correlations broadly concur with earlier findings, including those by Pekez [16], who also reported an age-related decline in performance on the DST, DICE, FFT, and EFT‑s.

Educational attainment was found to significantly correlate with performances on the CITY and VVT. It has been demonstrated that a higher level of education leads to improvements in learning and memory tasks, including the CITY [23, 24]. Although Hochrathner [15] reported an educational advantage in the CITY assessment, this study did not replicate the enhanced performance among individuals with higher education in other tasks such as the FACE, FFT, and DST.

The analysis of screening data revealed correlations with only a select number of subtests. Specifically, the VVT 3.0 exhibited a negative correlation with the FACE, and the BDI-II showed a negative correlation with both the EFT‑s round 2 and the VVT. The WST-IQ demonstrated a significant positive correlation with the VVT, aligning with expectations since the WST-IQ assesses verbal intelligence and the VVT measures language abilities.

Furthermore, intercorrelations were explored to determine whether subtests assessing the same cognitive domain were related. The DST and PCT showed significant correlations, indicating their alignment within the complex attention domain. Similarly, scores from the FACE and CITY were significantly correlated, reflecting the learning and memory domain. A correlation within the executive function domain was observed solely between the FFT and DICE. The absence of correlations in the language and social cognition domains was noted, with this outcome attributed to the sole use of the VVT for language measurement and the exclusive application of the EFT‑s for assessing social cognition capabilities. For a thorough and precise evaluation of the extent to which the subtest data represent the cognitive domains, further research with larger sample sizes and the application of principal component analysis is recommended.

This research was conducted as a pilot study without the implementation of formal sample size determination or power analysis. Consequently, the outcomes should not be generalized to the wider population. However, these initial findings are instrumental in guiding subsequent, more comprehensive research efforts and will facilitate the ongoing refinement of the INCP.
